# Enhancing the Evidence: Critical Insights and Future Directions for Texture and Color Enhancement Imaging in Colorectal Neoplasia Detection

**DOI:** 10.1002/jgh3.70342

**Published:** 2026-07-03

**Authors:** Anastasios Koulaouzidis, Wojciech Marlicz

**Affiliations:** ^1^ Department of Clinical Research University of Southern Denmark (SDU) Odense Denmark; ^2^ Department of Surgery Odense University Hospital (OUH) and Svendborg Hospital Svendborg Denmark; ^3^ Department of Gastroenterology Pomeranian Medical University (PUM) Szczecin Poland

Dear Editor,

We read with great interest the recent systematic review and meta‐analysis by Judge et al. [1] examining texture and color enhancement imaging (TXI) for colonic neoplasia detection. The authors provide an important synthesis of emerging evidence and suggest that TXI improves adenoma detection rate (ADR), polyp detection rate (PDR), and mean adenomas per patient (MAP) compared with white light imaging (WLI). The findings are encouraging, though several points merit further consideration before TXI can be regarded as a transformative advance in colorectal cancer (CRC) prevention.

Prior work, including a January 2025 published meta‐analysis [2] that reported clear benefits in ADR, had already begun to shape the field but omitted several important recent studies. The recently published, multicenter deTXIon randomized controlled trial (RCT) [3], confirmed TXI's superiority for selected detection endpoints. Emerging data combining TXI with endocuff vision suggested additive benefits [4]. A prospective crossover randomized trial (online ahead of print in August 2024), conducted between June 2022 and January 2023, found that TXI was associated with significant increases in PDR and ADR compared to WLI, especially in the right colon [5]. Incorporating these studies would likely refine pooled estimates and reduce reliance on smaller early series. Therefore, the July 2024 search cutoff limits completeness, as the analyzed body of evidence remains modest; just four studies with a total of 5481 patients, only two of which were RCTs. The largest contribution by Toyoshima et al. included 3940 patients but incorporated routine dye‐spray chromoendoscopy in both arms, a methodological choice that likely diluted the incremental effect of TXI [3]. Judge et al.'s sub‐analysis excluding this study, although not pre‐specified, reduced statistical heterogeneity and revealed stronger effect sizes, with the reported absolute ADR improvement increasing from ~8% to ~14%. While illuminating, this post hoc approach warrants caution regarding potential bias. Differences in study design, scope, and methodology (e.g., Sakamoto's right‐colon‐only crossover) further complicate comparability and underscore the fragility of the evidence base.

Some apparent inconsistencies in reporting also warrant clarification. The results section states that proximal lesion detection was not significant (*p* = 0.57; Section 3.2.3), which appears to contrast with the abstract and Figure 5, where a significant effect is reported (MD 0.21, 95% CI, 0.15–0.27, *p* < 0.001). Sessile serrated lesion (SSL) detection was significantly improved (RR 1.44; 95% CI, 1.02–2.02, *p* ≤ 0.01; Section 3.2.3), though the analysis remains underpowered, with inclusion of only three studies, with incomplete subgroup data on lesion size and morphology and without harmonization of lesion definitions or size thresholds. Outcomes for advanced adenomas and CRC were not reported in the included studies, despite their greater clinical relevance. Funnel plots and Egger's test indicate small‐study effects (*p* = 0.046; Section 3.3, Figure 8); with so few studies, risk of effect size overestimation remains, and additional approaches such as trim‐and‐fill analysis could have strengthened transparency. The impossibility of blinding endoscopists introduces operator bias, particularly in crossover or observational designs.

Even where statistical significance is observed, the clinical meaning is less certain. Many additional detected lesions were reported as diminutive adenomas or SSLs, whose contribution to long‐term CRC risk remains debated. While any ADR improvement is welcome, it is not yet clear whether the modest gains achieved with TXI translate into reductions in post‐colonoscopy CRC incidence or CRC‐related mortality. Without long‐term outcome data, TXI's role should be considered promising but not definitive. Questions of generalizability further temper enthusiasm. Three studies were Japan‐led or included Japan (Sakamoto, Toyoshima, Antonelli, multicenter with Italy/Germany/Japan), with one from Australia (Young), with high baseline ADRs, frequent use of dye‐spray chromoendoscopy, and varied indications (screening, surveillance, and symptomatic populations). These features limit applicability to Western practice, where ADRs are generally lower and technology adoption is more variable. In addition, all studies required the Olympus EVIS X1 platform, which, while increasingly available in tertiary centers, is not yet universal. Such factors highlight the importance of validating TXI's benefits in broader, real‐world settings.

Practical Implications for Clinicians: For endoscopists, TXI could enhance detection in high‐risk cases (e.g., flat polyps or SSLs), potentially integrating seamlessly with AI tools or distal attachments like endocuff vision for additive gains [4]. However, implementation should weigh costs (e.g., equipment upgrades) against benefits; a cost‐effectiveness analysis in diverse populations would be valuable. In routine practice, prioritize TXI in settings with suboptimal ADRs, but combine with quality metrics like withdrawal time to maximize impact.

Table 1 summarizes key methodological characteristics and principal findings of the three most recent meta‐analyses evaluating TXI in colorectal neoplasia detection. In summary, Judge et al. have provided a rigorous and valuable synthesis of the emerging evidence on TXI. Their work highlights the promise of this technology, particularly in enhancing sessile serrated lesion detection, while also underscoring the limitations of the current literature: heterogeneity, publication bias, incomplete reporting, and restricted generalizability. Further supporting these limitations, a subsequent meta‐analysis by Shahzil et al., online ahead of print in April 2025 [6] across GI lesions confirms TXI's benefits but highlights similar issues with study heterogeneity and restricted applicability to Western practice. Further multicenter, independently funded randomized trials, ideally including real‐world effectiveness studies, cost–benefit evaluations, and head‐to‐head comparisons with other image‐enhanced modalities (e.g., AI‐assisted systems), will be essential to define TXI's role. Until such evidence is available, TXI should be regarded as a promising adjunct within the expanding landscape of image‐enhanced endoscopy, rather than as an established standard of care.

**TABLE 1:** Comparison of study characteristics, search strategies, and key outcomes across recent meta‐analyses evaluating texture and color enhancement imaging (TXI) for colorectal neoplasia detection.

Aspect	Judge et al. (*JGH Open*, Aug 2025) [1]	Mitev et al. (*Endoscopy International Open*, Jan 2025) [2]	Shahzil et al. (*DEN Open*, online ahead of print in April 2025) [6]
Search Cutoff	July 1, 2024	May 2024	Not explicitly stated (searches up to ~Feb 2025 based on receipt date)
No. Studies Included (Colorectal Focus)	4 (all colorectal)	3 (all colorectal)	17 total (3 RCTs, 14 observational; 3–5 in colorectal subgroup)
No. Patients (Colorectal Focus)	5481	1541	16 634 total (~5000+ in colorectal subgroup based on overlaps)
ADR Effect	RR 1.24 (55.8% vs. 47.8%, *p* < 0.001); sub‐analysis excluding Toyoshima: absolute +14%	RR 1.32 (57.8% vs. 43.6%, 95% CI, 1.20–1.46, *p* < 0.001, I^2^ = 0%)	OR 1.66 (95% CI, 1.31–2.12, I^2^ = 0%; colorectal subgroup, moderate certainty)
APC/MAP Effect	MD 0.41 (*p* = 0.005); sub‐analysis: stronger effect	MD 0.50 (95% CI, 0.37–0.64, *p* < 0.001, I^2^ = 0%)	MD 0.48 (95% CI, 0.25–0.70, I^2^ = 0%; colorectal subgroup, moderate certainty)
Other Key Findings	PDR: RR 1.23 (*p* < 0.001); Proximal lesions: MD 0.21 (*p* < 0.001); SSL: RR 1.44 (*p* ≤ 0.01); No difference in withdrawal time; Small‐study bias noted	Superior for nonpolypoid/flat, proximal, ≥ 10 mm adenomas; Shorter withdrawal times; Focus on various indications (FIT+, surveillance, symptoms)	Overall lesion detection: OR 1.84 (95% CI, 1.52–2.22); Colorectal detection: OR 1.77; Visibility scores: MD 0.50; Color differentiation: MD 3.31; TXI Mode 1 > Mode 2; No endoscopist experience effect
Limitations Noted	Heterogeneity; Post hoc sub‐analysis; Japan bias (3/4 studies); Small evidence base; Operator bias; No advanced adenoma/CRC outcomes	Small number of studies (2 RCTs); Geographic bias (mostly Asia); Underpowered for subgroups; No long‐term outcomes	Heterogeneity; Geographic bias (mostly Japan); Small RCTs; Observational study dominance; Limited Western applicability; No direct comparisons to other IEE modalities
Certainty of Evidence	Not formally graded (narrative on fragility)	Not formally graded; Low heterogeneity (I^2^ = 0%)	GRADED: High for visibility/color; Moderate for detection/ADR (colorectal)

Abbreviations: ADR, adenoma detection rate; APC/MAP, adenomas per colonoscopy/mean adenomas per patient; CRC, colorectal cancer; IEE, image‐enhanced endoscopy; MD, mean difference; OR, odds ratio; RCT, randomized controlled trial; RR, risk ratio; SSL, sessile serrated lesion; TXI, texture and color enhancement imaging; WLI, white‐light imaging.

## Conflicts of Interest

A.K. and W.M. are or have been Guest editors for MDPI; A.K. and W.M. are Section board members of Diagnostics and JCM. A.K. is Associate Editor of the Annals of Gastroenterology and Therapeutic Advances of Gastrointestinal Endoscopy. A.K. is the immediate past president of the iCARE group. W.M. is Co‐editor in chief Przeglad Gastroenterologiczny/Gastroenterology Review/, President of the Polish Society of Gastroenterology, Chair of Train the Trainers and Governing Council WGO, Country Representative/Poland/—European Lifestyle Medicine Organization and Country Coordinator/Poland/—HpEuReg Registry.

## Data Availability

Data sharing not applicable to this article as no datasets were generated or analyzed during the current study.
